# Supplementary material for: Roles of Oxygen Vacancies of CeO2 and Mn-Doped CeO2 with the Same Morphology in Benzene Catalytic Oxidation
Source: Molecules. 2021 Oct 21;26(21):6363. doi: 10.3390/molecules26216363 (PMC8587142; doi:10.3390/molecules26216363)
Supplement: Supplementary file 1 [file molecules-26-06363-s001.zip › molecules-1403746-supplementary.pdf]

Supplementary Materials

Roles of Oxygen Vacancies of ceo2 and MN-Doped ceo2 With the Same Morphology in Benzene Catalytic Oxidation

Min Yang [1], Genli Shen [2], Qi Wang [2], Ke Deng [2], Mi Liu [2], Yunfa Chen [3], Yan Gong [2,*] and Zhen Wang [2,*]

1 School of Chemistry and Biological Engineering, University of Science and Technology Beijing, 100083, China; yangmin@ustb.edu.cn (M. Y.)
2 CAS Key Laboratory of Standardization and Measurement for Nanotechnology, CAS Center for Excellence in Nanoscience, National Center for Nanoscience and Technology, Beijing 100190, China; shengl@nanoctr.cn (G.S.); wangq@nanoctr.cn (Q.W.); kdeng@nanoctr.cn (K.D.); liumi@nanoctr.cn (M.L.)
3 State Key Laboratory of Multiphase Complex Systems, Institute of Process Engineering, Chinese Academy of Sciences, Beijing 100190, China; yfchen@home.ipe.ac.cn
* Correspondence: wangzh@nanoctr.cn (Z.W.); gongyan@nanoctr.cn (Y.G.); Tel.: +86-10-82545755; Fax: +86-10-62525716

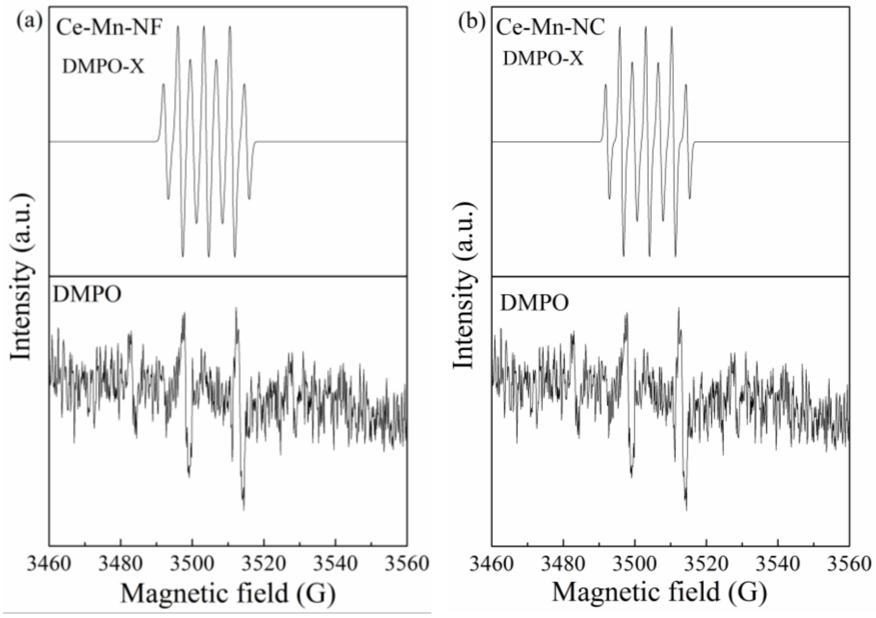

Figure S1. Electron paramagnetic resonance signals of DMPO-X (a): Ce-Mn-NF, (b): Ce-Mn-NC.

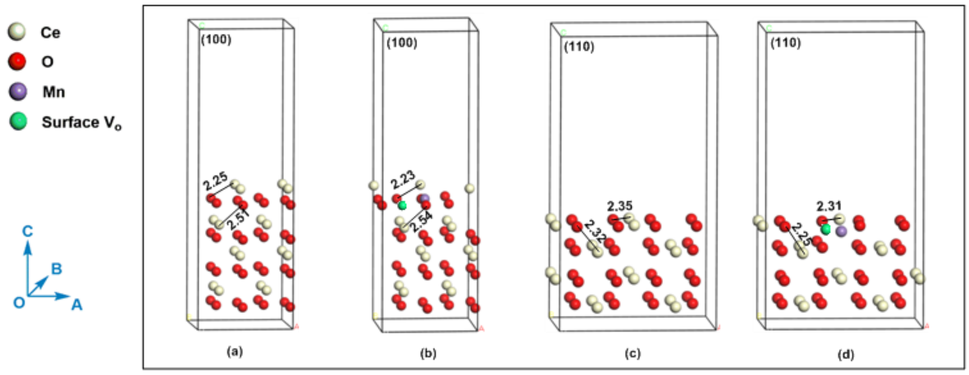

Figure S2. The distances of Ce-O on the surface and in bulk: (**a**): CeO_2 (100) plane, (**b**) Mn-doped CeO_2 (100) plane, (**c**): CeO_2 (110) plane, (**d**) Mn-doped CeO_2 (110) plane
